# Real-World Evaluation of the Immune Checkpoint Inhibitor Score (IChIS) in Advanced Non-Small Cell Lung Cancer Treated with Immunotherapy

**DOI:** 10.3390/jcm15197750

**Published:** 2026-10-08

**Authors:** İsmail Dilli, Engin Eren Kavak, Mehmetcan Atak, Selahattin Çelik, Özlem Aydın İsak, Esra Zeynelgil, Doğan Yazılıtaş

**Affiliations:** Department of Medical Oncology, Etlik City Hospital, 06170 Ankara, Turkey

**Keywords:** non-small cell lung cancer, immunotherapy, prognosis, IChIS, survival

## Abstract

**Background:** The Immune Checkpoint Inhibitor Score (IChIS), an inflammation-based biomarker incorporating neutrophil and immature granulocyte counts, was recently developed to predict outcomes in patients with advanced non-small cell lung cancer (NSCLC) receiving immune checkpoint inhibitors (ICIs). However, evidence regarding its prognostic performance in independent real-world populations remains limited. We aimed to evaluate the prognostic significance of IChIS in a real-world cohort of patients with advanced NSCLC treated with immunotherapy. **Methods:** This retrospective single-center cohort study included 169 patients with advanced, recurrent, or metastatic NSCLC who received ICIs with palliative intent between September 2022 and December 2025. IChIS ranged from 0 to 2 and was analyzed as a categorical variable, with IChIS 0 as the reference category. The primary endpoint was progression-free survival (PFS), while overall survival (OS) was evaluated as a secondary exploratory endpoint. Survival outcomes were estimated using the Kaplan–Meier method and evaluated using Cox proportional hazards regression. Multivariable models included ICI treatment line as a clinically relevant potential confounder. **Results:** A total of 169 patients were included. The median follow-up was 14.1 months. IChIS 0, 1, and 2 were observed in 105 (62.1%), 33 (19.5%), and 31 (18.4%) patients, respectively. Median PFS decreased across increasing IChIS categories. In multivariable Cox regression analysis, compared with IChIS 0, both IChIS 1 (adjusted HR 1.61, 95% CI 1.00–2.59; *p* = 0.04) and IChIS 2 (adjusted HR 3.63, 95% CI 2.22–5.92; *p* < 0.001) were independently associated with shorter PFS. For OS, IChIS 1 was not significantly associated with outcome (adjusted HR 1.51, 95% CI 0.86–2.64; *p* = 0.15), whereas IChIS 2 was independently associated with shorter OS (adjusted HR 4.40, 95% CI 2.53–7.64; *p* < 0.001). **Conclusions:** Higher IChIS was independently associated with shorter PFS in patients with advanced NSCLC receiving ICIs, supporting its potential role as a simple and readily available prognostic biomarker. The association with OS was primarily observed in patients with IChIS 2 and should be interpreted cautiously given the relatively short follow-up. Prospective multicenter validation is warranted.

## 1. Introduction

The introduction of immune checkpoint inhibitors (ICIs) has substantially changed the management of advanced non–small cell lung cancer (NSCLC), with a subset of patients achieving durable disease control and extended survival [1]. Nevertheless, treatment benefit varies considerably, and early progression remains frequent in clinical practice [2]. Identifying readily available biomarkers that can distinguish patients with different prognoses may therefore improve risk stratification and support clinical decision-making.

Programmed death-ligand 1 (PD-L1) expression remains an established tumor-based biomarker in the clinical use of ICIs, but it does not fully account for the substantial variability in patient outcomes [3]. Prognosis may also be influenced by host-related characteristics, including systemic inflammatory activity, which is closely linked to the interaction between the immune system and the tumor microenvironment [4]. Accordingly, inflammatory parameters derived from routine peripheral blood testing have attracted interest as accessible markers of the host–tumor relationship across several malignancies [5].

Kang et al. [6] recently developed and prospectively validated the Immune Checkpoint Inhibitor Score (IChIS), which combines neutrophil and immature granulocyte counts to provide a simple measure of systemic inflammatory status in patients with NSCLC treated with ICIs. Whether its prognostic performance is reproducible in independent real-world cohorts remains insufficiently established. We therefore evaluated the association between IChIS and survival outcomes in a real-world cohort of patients with advanced NSCLC receiving ICIs, considering PFS as the primary endpoint and OS as a secondary exploratory endpoint.

## 2. Materials and Methods

### 2.1. Study Design and Patients

This retrospective, single-center cohort study included consecutive patients with histologically confirmed advanced non-small cell lung cancer (NSCLC) who received immune checkpoint inhibitors (ICIs) with palliative intent at Ankara Etlik City Hospital between September 2022 and December 2025. Consecutive eligible patients were identified through institutional electronic medical records. Patients treated with ICIs in the neoadjuvant, adjuvant, or consolidation setting were excluded. Patients initially diagnosed with stage I–III disease were eligible only if they subsequently developed recurrent or metastatic disease and received ICI therapy with palliative intent. Accordingly, all patients had advanced, recurrent, or metastatic NSCLC at the time of ICI initiation. Disease stage reported in this study refers to the stage at initial NSCLC diagnosis. Additional exclusion criteria included incomplete baseline laboratory data or missing follow-up information. All eligible patients meeting the inclusion criteria during the study period were included.

### 2.2. Data Collection

Clinical, pathological, and laboratory data were obtained from electronic medical records. Baseline variables included age, sex, smoking status, ECOG performance status, histologic subtype, disease stage, presence of brain metastases, driver mutation status, PD-L1 expression, immunotherapy regimen, and line of ICI therapy. The line of ICI therapy was defined according to systemic treatment received in the advanced or recurrent disease setting. Prior neoadjuvant, adjuvant, or definitive chemoradiotherapy was not considered a prior line of therapy for advanced disease. ICI treatment was categorized as first-line or later-line (≥2). All patients received ICI as monotherapy, and no patient received concurrent cytotoxic chemotherapy at the time of ICI initiation. Baseline laboratory values were defined as measurements obtained within 7 days prior to initiation of immunotherapy.

### 2.3. Definition of IChIS

The IChIS was calculated according to the original definition proposed by Kang et al. using two systemic inflammatory parameters: neutrophil count and immature granulocyte count. One point was assigned for each of the following: neutrophil count >9000 cells/µL and immature granulocyte count >80 cells/µL. The total IChIS ranged from 0 to 2, with higher scores indicating a greater degree of systemic inflammation (Table 1).

### 2.4. Outcomes

The primary endpoint was PFS, with OS considered a secondary exploratory endpoint. PFS was measured from ICI initiation until radiologically or clinically documented progression or death from any cause, whichever occurred first; patients without a PFS event were censored at their last disease assessment. OS was measured from ICI initiation until death from any cause, with surviving patients censored at their most recent follow-up. Events occurring within the first 3 months after ICI initiation were retained in the survival analyses.

### 2.5. Statistical Analysis

Statistical analyses were performed using BlueSky Statistics (version 10.3.2; BlueSky Statistics LLC, Chicago, IL, USA). Continuous data were described using either mean ± standard deviation or median with interquartile range, depending on their distribution; categorical data were summarized as counts and percentages.

Time-to-event distributions were characterized with Kaplan–Meier estimates, with between-group differences assessed by the log-rank test. The duration of follow-up was quantified using the reverse Kaplan–Meier approach. Associations between candidate variables and survival outcomes were examined using univariable and multivariable Cox proportional hazards models. Multivariable models were specified a priori using clinically relevant covariates rather than a *p*-value-based variable-selection strategy. IChIS was modeled as a categorical variable, with IChIS 0 as the reference category. Multivariable models included clinically relevant covariates according to the outcome of interest. The PFS model included ECOG performance status, BMI, ICI treatment line, and PD-L1 expression, whereas the OS model included ECOG performance status, sex, ICI treatment line, and PD-L1 expression. Effect estimates from the Cox models are presented as hazard ratios (HRs) with corresponding 95% confidence intervals (CIs). Model proportionality was examined using tests based on Schoenfeld residuals. Potential effect modification by ICI treatment line was assessed by including an IChIS × ICI treatment-line interaction term in the Cox regression models. A two-sided *p* value < 0.05 was considered statistically significant.

Missingness was examined across all baseline variables. Although missingness was below 5% overall, the covariates entered into the revised multivariable Cox models were complete for all 169 patients. Consequently, these models used the full cohort and no multiple imputation was necessary.

## 3. Results

### 3.1. Patient Characteristics

A total of 169 patients with advanced non–small cell lung cancer (NSCLC) receiving palliative immunotherapy were included. The mean age was 64.3 ± 8.1 years, and the majority were male (85.2%). Most patients were current or former smokers (89.4%), with a median smoking exposure of 40 pack-years (IQR, 30–150).

Comorbidities were present in 63.9% of patients, with 33.8% having two or more comorbid conditions. The median body mass index (BMI) was 24.9 kg/m^2^ (IQR, 22.0–39.5). ECOG performance status was 0 in 39.0%, 1 in 47.9%, and 2 in 13.1% of patients.

Adenocarcinoma was the predominant histology (62.1%). At diagnosis, 60.9% of patients had stage IV disease, and brain metastases were present in 11.8%. Driver alterations were identified in 20 patients (11.8%), all of which were KRAS mutations; no EGFR mutations or ALK rearrangements were identified in the cohort. PD-L1 expression was <1% in 56.2%, 1–49% in 23.1%, and ≥50% in 20.7%. Nivolumab was the predominant ICI administered (91.7%). All patients received ICI monotherapy without concurrent cytotoxic chemotherapy (Table 2).

### 3.2. Baseline Laboratory and IChIS Distribution

Baseline laboratory parameters are summarized in Table 3. The median LDH level was 205 U/L (IQR, 178–260), and the median albumin level was 40.6 g/dL (IQR, 37.6–42.9). Median neutrophil and immature granulocyte counts were 5740 (IQR, 4750–7750) and 40 cells/µL (IQR, 20–60), respectively.

Based on predefined cut-off values, elevated neutrophil and immature granulocyte scores were observed in 31.4% and 34.3% of patients, respectively. According to IChIS classification, 105 patients (62.1%) were categorized as IChIS 0, 33 (19.5%) as IChIS 1, and 31 (18.4%) as IChIS 2.

### 3.3. Survival Outcomes in the Overall Cohort

After a median follow-up of 14.1 months, the median PFS and overall OS in the overall cohort were 4.6 months (95% CI, 3.7–6.1) and 10.4 months (95% CI, 7.3–14.2), respectively (Figure 1).

Survival outcomes differed significantly according to IChIS category (log-rank *p* < 0.001). A clear stepwise decline in survival was observed with increasing IChIS. Median PFS was 5.5 months for IChIS 0, 3.6 months for IChIS 1, and 3.0 months for IChIS 2 (Figure 2). Similarly, median OS was 13.5, 10.7, and 5.8 months for IChIS 0, 1, and 2, respectively (Figure 3).

### 3.4. Cox Regression Analyses

In univariate Cox regression analysis, compared with IChIS 0, both IChIS 1 (HR 1.72, 95% CI 1.09–2.71; *p* = 0.02) and IChIS 2 (HR 3.55, 95% CI 2.18–5.79; *p* < 0.001) were significantly associated with shorter PFS. In the multivariable model adjusted for ECOG performance status, BMI, ICI treatment line, and PD-L1 expression, both IChIS 1 (adjusted HR 1.61, 95% CI 1.00–2.59; *p* = 0.04) and IChIS 2 (adjusted HR 3.63, 95% CI 2.22–5.92; *p* < 0.001) remained independently associated with shorter PFS. ICI treatment line was not independently associated with PFS (later-line [≥2] vs. first-line: adjusted HR 1.35, 95% CI 0.90–2.01; *p* = 0.146) (Table 4).

In univariate Cox regression analysis, compared with IChIS 0, IChIS 1 was not significantly associated with OS (HR 1.47, 95% CI 0.85–2.56; *p* = 0.17), whereas IChIS 2 was significantly associated with shorter OS (HR 3.96, 95% CI 2.30–6.80; *p* < 0.001). In the multivariable model adjusted for ECOG performance status, sex, ICI treatment line, and PD-L1 expression, IChIS 1 remained non-significant (adjusted HR 1.51, 95% CI 0.86–2.64; *p* = 0.15), whereas IChIS 2 remained independently associated with shorter OS (adjusted HR 4.40, 95% CI 2.53–7.64; *p* < 0.001). ICI treatment line was not independently associated with OS (later-line [≥2] vs. first-line: adjusted HR 1.38, 95% CI 0.86–2.21; *p* = 0.18) (Table 5). Given the relatively short follow-up, the OS findings should be interpreted as exploratory.

The proportional hazards assumption was not violated for either multivariable Cox model based on Schoenfeld residual tests. For the PFS model, the global test was non-significant (*p* = 0.256), and no individual covariate showed evidence of violation (IChIS, *p* = 0.377; ECOG performance status, *p* = 0.208; ICI treatment line, *p* = 0.548; PD-L1 expression, *p* = 0.206; and BMI, *p* = 0.147). Similarly, for the OS model, the global test was non-significant (*p* = 0.652), with no evidence of violation for the individual covariates (IChIS, *p* = 0.703; ECOG performance status, *p* = 0.605; ICI treatment line, *p* = 0.743; PD-L1 expression, *p* = 0.572; and sex, *p* = 0.096). No significant interaction was observed between IChIS and ICI treatment line for either PFS or OS (*p* for interaction = 0.640 and *p* = 0.639, respectively), providing no evidence that the association between IChIS and survival differed according to treatment line.

## 4. Discussion

In this retrospective real-world cohort of patients with advanced NSCLC receiving immune checkpoint inhibitors, higher IChIS was independently associated with shorter PFS. Compared with IChIS 0, both IChIS 1 (adjusted HR 1.61, 95% CI 1.00–2.59; *p* = 0.04) and IChIS 2 (adjusted HR 3.63, 95% CI 2.22–5.92; *p* < 0.001) were independently associated with shorter PFS after adjustment for clinically relevant covariates. For OS, IChIS 1 was not significantly associated with outcome (adjusted HR 1.51, 95% CI 0.86–2.64; *p* = 0.15), whereas IChIS 2 remained independently associated with shorter OS (adjusted HR 4.40, 95% CI 2.53–7.64; *p* < 0.001). Given the relatively short follow-up, the OS findings should be interpreted as exploratory.

Our results add to previous evidence linking the host inflammatory state with outcomes during immune checkpoint inhibition [4,7,8]. Although PD-L1 expression provides clinically useful tumor-related information, it does not capture the full heterogeneity of outcomes observed with ICI therapy. Blood-based inflammatory measures may offer complementary information by reflecting systemic aspects of the host–tumor interaction. In our cohort, the association of higher IChIS categories with shorter PFS, together with the association between IChIS 2 and shorter OS, suggests that this readily available measure of systemic inflammation may provide prognostic information beyond conventional tumor-centered assessment.

The present results are directionally consistent with the original study by Kang et al. [6], in which IChIS was developed and prospectively validated among patients with advanced NSCLC treated with ICIs. Higher scores in that cohort identified patients with less favorable survival outcomes. Importantly, our study evaluated the same predefined score in a separate real-world population without re-deriving its components or thresholds. The observed association, particularly for PFS, therefore provides additional evidence that the prognostic information captured by IChIS may extend beyond its original development cohort.

We retained the neutrophil (>9000 cells/µL) and immature granulocyte (>80 cells/µL) thresholds specified in the original IChIS study rather than deriving new cut-offs from our dataset. This approach preserves the independent nature of the present evaluation and limits data-driven optimization; however, identical thresholds may not perform uniformly across populations or laboratory platforms. Their reproducibility should therefore be examined in larger multicenter cohorts representing different demographic groups and laboratory settings.

Importantly, the association between IChIS and PFS persisted after adjustment for ECOG performance status, BMI, ICI treatment line, and PD-L1 expression, with both IChIS 1 and IChIS 2 associated with shorter PFS compared with IChIS 0. For OS, after adjustment for ECOG performance status, sex, ICI treatment line, and PD-L1 expression, the association was primarily driven by IChIS 2, whereas IChIS 1 was not significantly associated with OS. These findings suggest a gradient of prognostic risk across IChIS categories, particularly for PFS, while the OS results require cautious interpretation given the limited follow-up.

PD-L1 expression is routinely considered when selecting systemic treatment for advanced NSCLC; however, PD-L1 ≥ 50% was not associated with either PFS or OS in our cohort. The absence of a significant association may partly reflect the clinical heterogeneity of this real-world population and suggests that host-derived inflammatory measures such as IChIS could provide prognostic information complementary to tumor PD-L1 status [9,10,11].

A practical advantage of IChIS is that it relies only on neutrophil and immature granulocyte counts obtained from routine blood testing and therefore requires no additional molecular assay. Its prognostic association persisted after accounting for ICI treatment line, suggesting that the information captured by the score was not solely attributable to differences in treatment sequencing. These characteristics make IChIS potentially suitable for baseline risk assessment using data already available in routine care. Nevertheless, its clinical role remains investigational, particularly because the OS analysis was exploratory. Prospective multicenter evaluation with longer follow-up is required before IChIS can be incorporated into routine prognostic assessment.

Several aspects of the study design should be considered when interpreting these findings. The retrospective experience of a single center is susceptible to selection effects and may not represent patient populations treated in other settings. Multivariable modeling can address measured covariates but cannot eliminate confounding from unrecorded clinical characteristics or differences in patient selection and treatment. Molecular characterization was also limited, restricting our ability to examine whether the prognostic association of IChIS varies across specific oncogenic subgroups. Furthermore, treatment heterogeneity inherent to real-world clinical practice, including differences in immunotherapy regimens, may have influenced outcomes. Because 66.9% of the cohort received ICIs in the later-line setting, prior systemic therapies may have influenced baseline inflammatory profiles, including neutrophil and immature granulocyte counts. Treatment-related myelosuppression, recovery from previous therapy, or changes in systemic inflammatory status may therefore affect IChIS measurements. Although treatment line was included in the multivariable models and no significant IChIS × treatment-line interaction was observed for PFS or OS, residual effects of prior therapies on baseline hematologic parameters cannot be completely excluded. Importantly, the present study supports IChIS as a prognostic marker in patients receiving ICI therapy rather than as a predictive biomarker of ICI benefit. Because all patients in this cohort received ICIs and no non-ICI comparator group was available, the study cannot determine whether IChIS predicts differential benefit from immunotherapy compared with other systemic treatments. In addition, the median follow-up of 14.1 months, although adequate for evaluating PFS, limits the maturity and robustness of the OS analysis. Therefore, the observed association between IChIS and OS should be considered exploratory and interpreted with caution until confirmed with longer follow-up. Future studies should prospectively validate IChIS in larger multicenter populations with longer follow-up and evaluate its performance across different treatment lines, laboratory platforms, and molecular subgroups. Such studies could also determine whether integrating IChIS with established clinical and tumor-based factors improves prognostic stratification beyond either approach alone.

## 5. Conclusions

IChIS was independently associated with PFS in patients with advanced NSCLC receiving immune checkpoint inhibitors and may represent a simple, readily available inflammation-based prognostic biomarker. For OS, the association was primarily observed in patients with IChIS 2, whereas IChIS 1 was not significantly associated with outcome. Given the relatively short follow-up, the OS findings should be considered exploratory. Prospective multicenter studies with longer follow-up are warranted to confirm these findings and establish the potential role of IChIS in prognostic risk stratification.

## Figures and Tables

**Figure 1:**
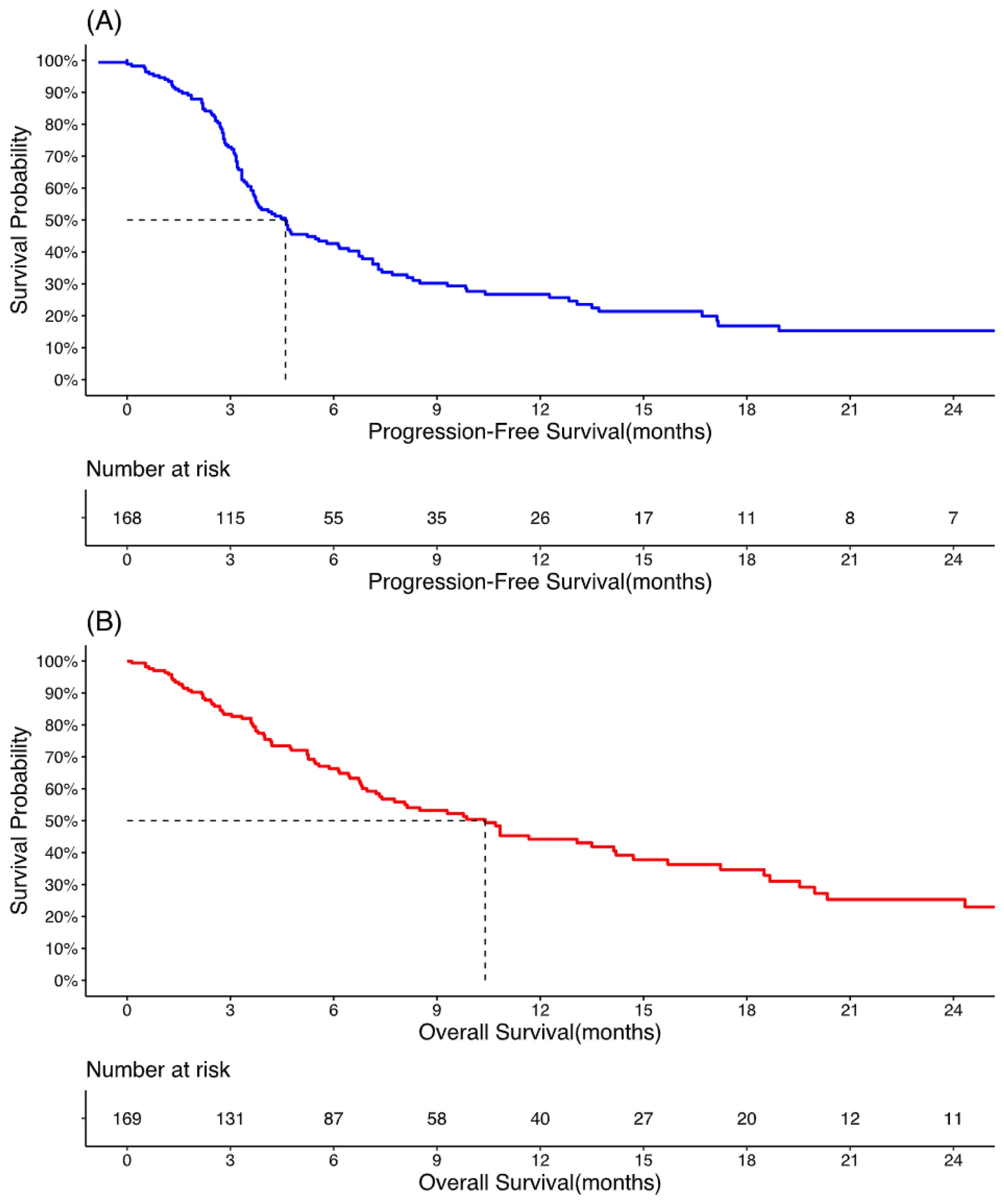
Dashed lines indicate the median survival estimates. Kaplan–Meier curves for progression-free survival (**A**)and overall survival (**B**) in the total cohort.

**Figure 2:**
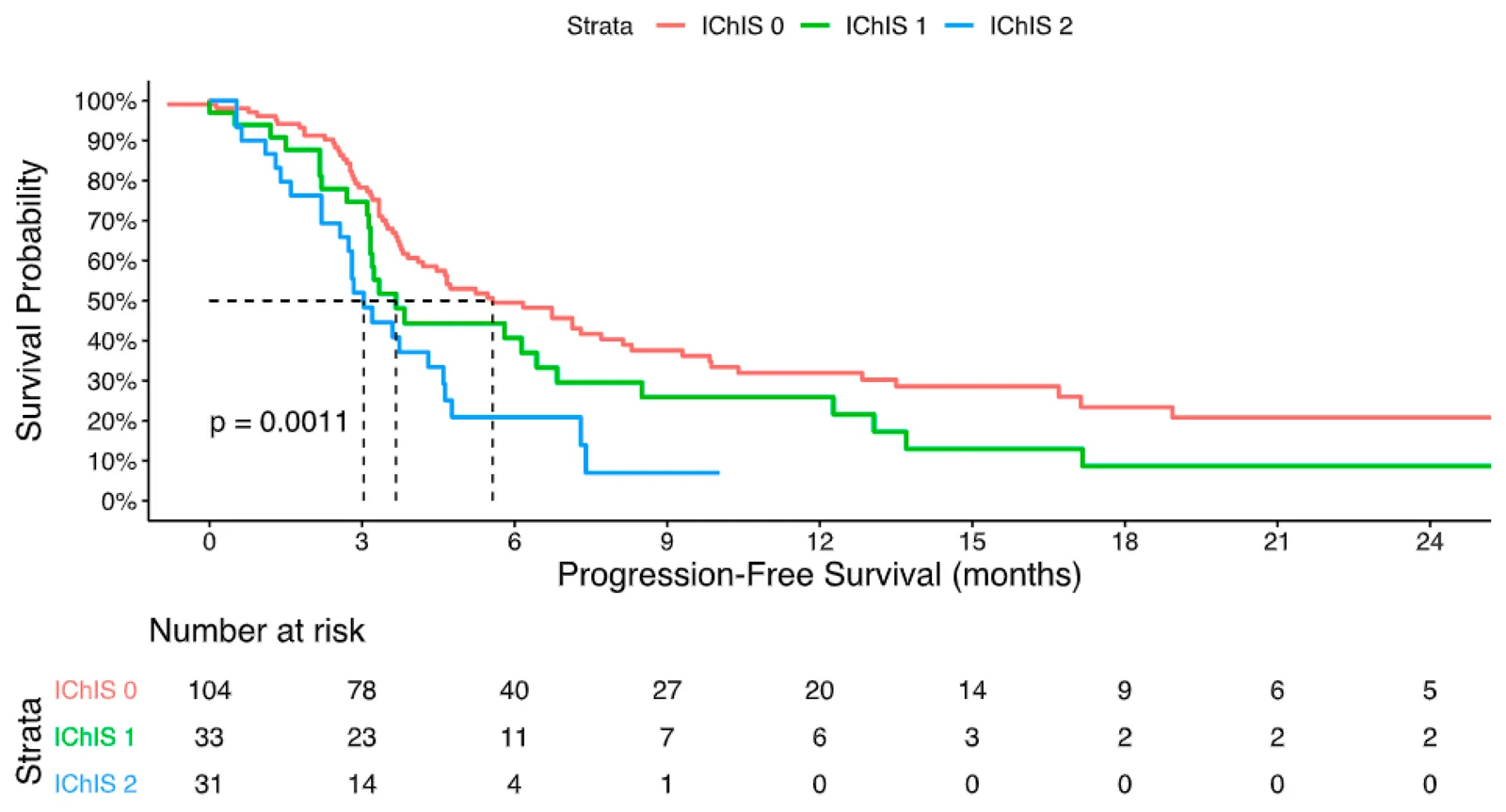
Dashed lines indicate the median PFS estimates for each IChIS category. Kaplan–Meier curves for PFS according to IChIS.

**Figure 3:**
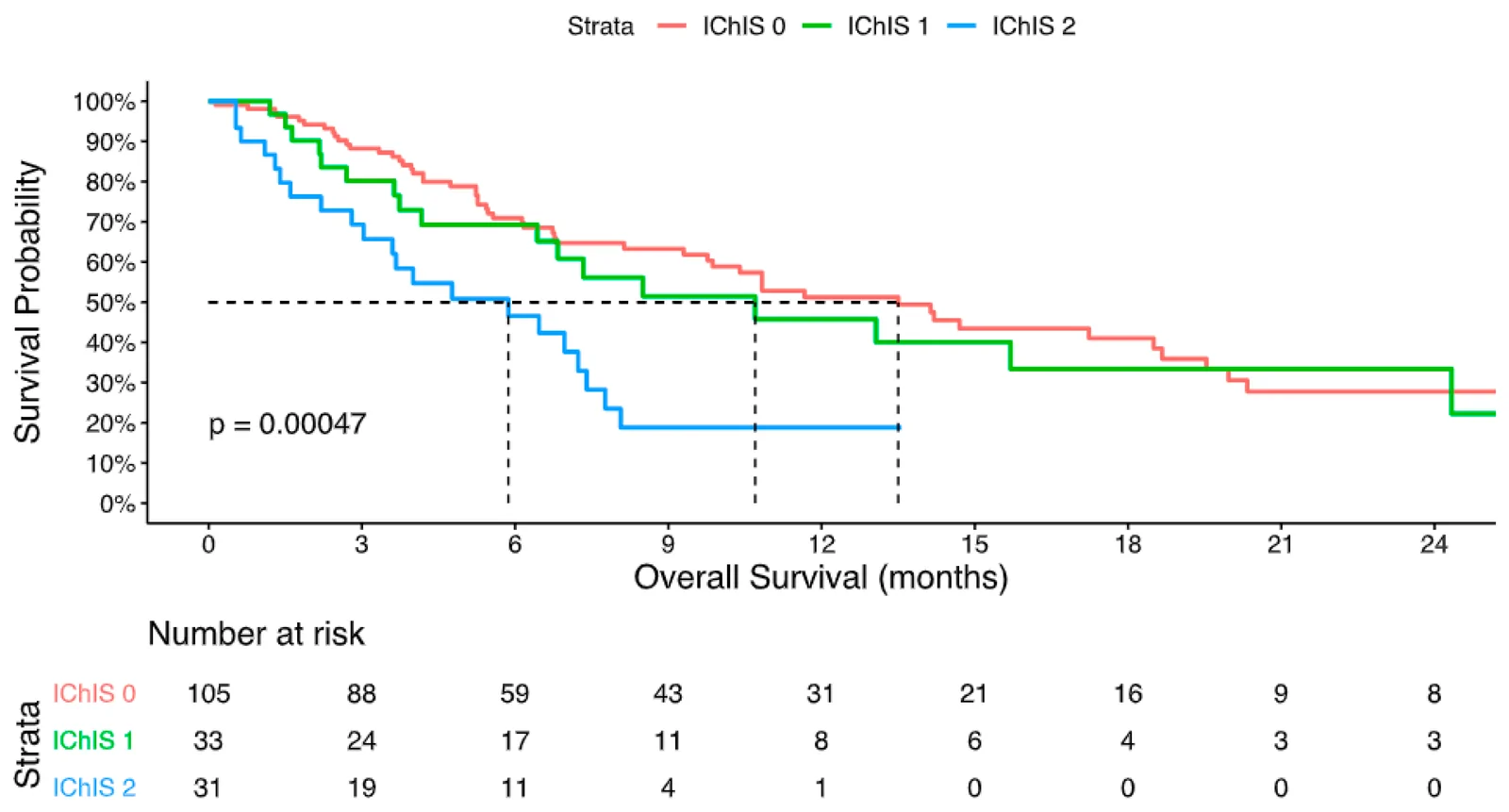
Dashed lines indicate the median OS estimates for each IChIS category. Kaplan–Meier curves for OS according to IChIS.

**Table 1:** The immune checkpoint inhibitor score (IChIS).

Parameter	Cut-Off	Score
Neutrophil count (cells/µL)	>9000	1
Immature granulocyte count (cells/µL)	>80	1
Total IChIS was defined as the sum of the two parameters (range: 0–2).		

**Table 2:** Baseline characteristics of patients receiving palliative immunotherapy (*n* = 169).

Age, mean ± S.D.	64.3 ± 8.1
Sex, n (%)	
Male	144 (85.2)
Female	25 (14.8)
Smoking, n (%)	
Never	18 (10.6)
Former/Current	151 (89.4)
Smoking exposure (pack-years), median (IQR)	40.0 (30.0–150.0)
Comorbidity, n (%)	
No	61 (36.1)
Yes	108 (63.9)
Number of comorbidities, n (%)	
0	59 (34.9)
1	53 (31.3)
≥2	57 (33.8)
Body mass index, kg/m^2^, median (IQR)	24.9 (22.0–39.5)
Body mass index category, n (%)	
<25 kg/m^2^	85 (50.3)
25–29.9 kg/m^2^	61 (36.1)
≥30 kg/m^2^	23 (13.6)
ECOG performance status	
0	66 (39.0)
1	81 (47.9)
2	22 (13.1)
Histology, n (%)	
Adenocarcinoma	105 (62.1)
Squamous	64 (37.9)
Disease stage at diagnosis, n (%)	
IB	1 (0.6)
IIA	1 (0.6)
IIB	8 (4.7)
IIIA	15 (8.9)
IIIB	22 (13.0)
IIIC	19 (11.2)
IVA	31 (18.3)
IVB	72 (42.6)
Brain metastasis, n (%)	
No	149 (88.2)
Yes	20 (11.8)
Driver mutation, n (%)	
No	149 (88.2)
Yes	20 (11.8)
Detected driver alteration, n (%)	
KRAS mutation	20 (11.8)
EGFR mutation	0 (0)
ALK rearrangement	0 (0)
KRAS mutation, n (%)	
KRAS G12C	12 (7.2)
KRAS G12V	1 (0.6)
KRAS G12X	5 (3.0)
KRAS G13X	1 (0.6)
KRAS C175G	1 (0.6)
PD-L1 expression (tumor proportion score), n (%)	
<1%	95 (56.2)
1–49%	39 (23.1)
≥50%	35 (20.7)
Immunotherapy Agent, n (%)	
Nivolumab	155 (91.7)
Pembrolizumab	7 (4.1)
Atezolizumab	6 (3.5)
Tislelizumab	1 (0.6)
Line of ICI therapy in the advanced/recurrent setting, n (%)	
First-line	56 (33.1)
Later-line (≥2)	113 (66.9)

Abbreviations: SD, standard deviation; IQR, interquartile range; ECOG, Eastern Cooperative Oncology Group; PD-L1, programmed death-ligand 1; ICI, immune checkpoint inhibitor; KRAS, Kirsten rat sarcoma viral oncogene homolog.

**Table 3:** Baseline laboratory and inflammatory parameters.

LDH, U/L, median (IQR)	205.0 (178.0–260.0)
Albumin, g/dL, median (IQR)	40.6 (37.6–42.9)
Hemoglobin, g/dL, median (IQR)	12.9 (11.6–14.1)
Neutrophil count, cells/µL, median (IQR)	5740 (4750–7750)
Immature granulocyte count, cells/µL, median (IQR)	40.0 (20.0–60.0)
Neutrophil score (>9000 cells/µL), n (%)	53 (31.4)
Immature granulocyte score (>80 cells/µL), n (%)	58 (34.3)
Immune checkpoint inhibitor score (IChIS), n (%)	
0	105 (62.1)
1	33 (19.5)
2	31 (18.4)

**Table 4:** Univariate and multivariate Cox regression analyses for Progression-Free survival.

Variable	Univariate		Multivariate	
	HR (95% CI)	*p*-Value	HR (95% CI)	*p*-Value
IChIS 1 vs. 0	1.72 (1.09–2.71)	0.02	1.61 (1.00–2.59)	0.04
IChIS 2 vs. 0	3.55 (2.18–5.79)	<0.001	3.63 (2.22–5.92)	<0.001
ECOG performance status (≥2 vs. 0–1)	1.55 (1.0–2.54)	0.05	1.00 (0.68–1.46)	0.98
Brain metastasis (yes vs. no)	1.29 (0.75–2.24)	0.34		
PD-L1 expression (≥50% vs. <50%)	0.73 (0.46–1.17)	0.19	0.98 (0.61–1.58)	0.93
Histology (squamous vs. adenocarcinoma)	0.81 (0.56–1.18)	0.28		
Smoking status (ever vs. never)	1.26 (0.67–2.35)	0.46		
Body mass index (per 1 kg/m^2^ increase)	1.04 (1.0–1.08)	0.04	1.02 (0.98–1.06)	0.34
Sex (male vs. female)	0.84 (0.51–1.38)	0.51		
ICI treatment line (later-line [≥2] vs. first-line)	1.29 (0.87–1.91)	0.212	1.35 (0.90–2.01)	0.146

Hazard ratios (HRs) and 95% confidence intervals (CIs) were estimated using Cox proportional hazards regression models. IChIS was modeled as a categorical variable, with IChIS 0 as the reference category. The multivariable model included ECOG performance status, BMI, ICI treatment line, and PD-L1 expression as clinically relevant covariates.

**Table 5:** Univariate and multivariate Cox regression analyses for overall survival.

Variable	Univariate		Multivariate	
	HR (95% CI)	*p*-Value	HR (95% CI)	*p*-Value
IChIS 1 vs. 0	1.47 (0.85–2.56)	0.17	1.51 (0.86–2.64)	0.15
IChIS 2 vs. 0	3.96 (2.30–6.80)	<0.001	4.40 (2.53–7.64)	<0.001
ECOG performance status (≥2 vs. 0–1)	2.21 (1.33–3.69)	0.002	1.13 (0.72–1.76)	0.60
Brain metastasis (yes vs. no)	1.02 (0.55–1.88)	0.93		
PD-L1 expression (≥50% vs. <50%)	0.66 (0.39–1.13)	0.13	0.97 (0.56–1.66)	0.91
Histology (squamous vs. adenocarcinoma)	0.80 (0.52–1.23)	0.31		
Smoking status (ever vs. never)	1.30 (0.62–2.70)	0.47		
Body mass index (per 1 kg/m^2^ increase)	1.01 (0.97–1.06)	0.41		
Sex (male vs. female)	0.60 (0.35–1.00)	0.05	0.62 (0.36–1.05)	0.08
ICI treatment line (later-line [≥2] vs. first-line)	1.37 (0.86–2.17)	0.18	1.38 (0.86–2.21)	0.18

Hazard ratios (HRs) and 95% confidence intervals (CIs) were estimated using Cox proportional hazards regression models. IChIS was modeled as a categorical variable, with IChIS 0 as the reference category. The multivariable model included ECOG performance status, sex, ICI treatment line, and PD-L1 expression as clinically relevant covariates.

## Data Availability

The datasets generated and/or analyzed during the current study are available from the corresponding author on reasonable request.

## References

[1] Khan S., Upadhyay S., Kauser S., Hasan G.M., Lu W., Waters M., Hassan I., Sohal S.S. (2025). Redefining the Diagnostic and Therapeutic Landscape of Non-Small Cell Lung Cancer in the Era of Precision Medicine. J. Clin. Med..

[2] Gang X., Yan J., Li X., Shi S., Xu L., Liu R., Cai L., Li H., Zhao M. (2024). Immune checkpoint inhibitors rechallenge in non-small cell lung cancer: Current evidence and future directions. Cancer Lett..

[3] Incorvaia L., Fanale D., Badalamenti G., Barraco N., Bono M., Corsini L.R., Galvano A., Gristina V., Listì A., Vieni S. (2019). Programmed Death Ligand 1 (PD-L1) as a Predictive Biomarker for Pembrolizumab Therapy in Patients with Advanced Non-Small-Cell Lung Cancer (NSCLC). Adv. Ther..

[4] Santo V., Brunetti L., Pecci F., Peroni M., Barnini G., Paoloni F., Buti S., Tiseo M., Ricciuti B., Pinato D.J. (2025). Host-related Determinants of Response to Immunotherapy in Non-small Cell Lung Cancer: The Interplay of Body Composition, Metabolism, Sex and Immune Regulation. Curr. Oncol. Rep..

[5] Rebuzzi S.E., Prelaj A., Friedlaender A., Cortellini A., Addeo A., Genova C., Naqash A.R., Auclin E., Mezquita L., Banna G.L. (2022). Prognostic scores including peripheral blood-derived inflammatory indices in patients with advanced non-small-cell lung cancer treated with immune checkpoint inhibitors. Crit. Rev. Oncol. Hematol..

[6] Kang D.H., Choi C.-M., Park C.-K., Oh I.-J., Kim Y.-C., Yoon S.H., Kim Y., Lee J.E. (2024). Immune Checkpoint Inhibitor Score Predicts Survival Benefit of Immunotherapy in Patients with Non-small Cell Lung Cancer. Tuberc. Respir. Dis..

[7] Baci D., Cekani E., Imperatori A., Ribatti D., Mortara L. (2022). Host-Related Factors as Targetable Drivers of Immunotherapy Response in Non-Small Cell Lung Cancer Patients. Front. Immunol..

[8] Vornicu V., Negru A.-G., Vonica R.C., Cosma A.A., Pasca-Fenesan M.M., Cimpean A.M. (2026). Inflammatory–Molecular Clusters as Predictors of Immunotherapy Response in Advanced Non-Small-Cell Lung Cancer. J. Clin. Med..

[9] Vrankar M., Kern I., Stanic K. (2020). Prognostic value of PD-L1 expression in patients with unresectable stage III non-small cell lung cancer treated with chemoradiotherapy. Radiat. Oncol..

[10] Usluer O., Gökbayrak Ö.E., Erol A., Aktaş T.Ç., Batıhan G., Kaya Ş.Ö., Üçvet A., Aydoğdu Z., Altun Z., Öztop I. (2024). The prognostic significance of PD-1 and its ligands in non-small cell lung cancer. Turk. J. Thorac. Cardiovasc. Surg..

[11] Davis A.A., Patel V.G. (2019). The role of PD-L1 expression as a predictive biomarker: An analysis of all US Food and Drug Administration (FDA) approvals of immune checkpoint inhibitors. J. Immunother. Cancer.

